# Nifedipine–pyrazine (2/1)

**DOI:** 10.1107/S1600536810031703

**Published:** 2010-08-11

**Authors:** Nate Schultheiss, Melanie Roe, Jared P. Smit

**Affiliations:** a SSCI (a division of Aptuit), 3065 Kent Avenue, West Lafayette, IN 47909, USA

## Abstract

In the title compound, 2C_17_H_18_N_2_O_6_·C_4_H_4_N_2_ [systematic name: 3,5-dimethyl 2,6-dimethyl-4-(2-nitro­phen­yl)-1,4-di­hydro­pyridine-3,5-dicarboxyl­ate–pyrazine (2/1)], the complete pyrazine molecule is generated by crystallographic inversion symmetry. The center of the pyrazine ring lies on an inversion center. The nifedipine mol­ecules are linked into chains along the *c* axis through N—H⋯O hydrogen bonds, while the pyrazine mol­ecules are organized in the structure through van der Waals inter­actions.

## Related literature

Co-crystalline materials are of pharmaceutical inter­est due to their ability to alter the physicochemical properties of active pharmaceutical ingredients (APIs) (Schultheiss *et al.*, 2009 ▶) and provide drug repositioning or life-cycle management (Trask, 2007 ▶). The corresponding crystal structure of nifedipine has been reported (Triggle *et al.*, 2003 ▶) and it also forms chains through N—H⋯O hydrogen bonds. Other crystalline forms also exist: polymorphs (Burger *et al.*, 1996 ▶) solvates/hydrates (Caira *et al.*, 2003 ▶) and a metal complex (Bontchev *et al.*, 2003 ▶), as well as a non-crystalline, amorphous phase (Miyazaki *et al.*, 2007 ▶).
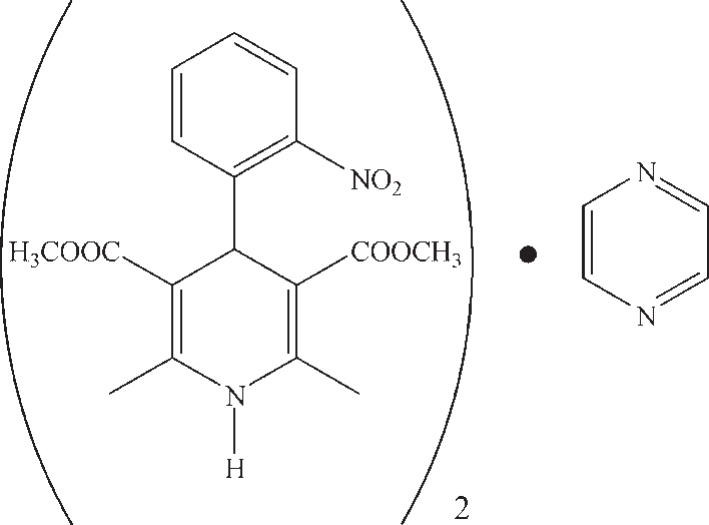


## Experimental

### 

#### Crystal data

C_19_H_20_N_3_O_6_
*M* *_r_* = 386.38
Monoclinic, 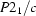

*a* = 13.6278 (14) Å
*b* = 9.1594 (9) Å
*c* = 14.4432 (14) Å
β = 94.841 (4)°
*V* = 1796.4 (3) Å^3^
*Z* = 4
Mo *K*α radiation
μ = 0.11 mm^−1^
*T* = 120 K
0.24 × 0.18 × 0.10 mm

#### Data collection

Bruker APEXII CCD diffractometer
27572 measured reflections
6070 independent reflections
4916 reflections with *I* > 2σ(*I*)
*R*_int_ = 0.036

#### Refinement

*R*[*F* ^2^ > 2σ(*F* ^2^)] = 0.043
*wR*(*F* ^2^) = 0.125
*S* = 1.07
6070 reflections
261 parameters
H atoms treated by a mixture of independent and constrained refinement
Δρ_max_ = 0.48 e Å^−3^
Δρ_min_ = −0.24 e Å^−3^

### 

Data collection: *APEX2* (Bruker, 2007 ▶); cell refinement: *SAINT* (Bruker, 2007 ▶); data reduction: *SAINT*; program(s) used to solve structure: *SHELXS97* (Sheldrick, 2008 ▶); program(s) used to refine structure: *SHELXL97* (Sheldrick, 2008 ▶); molecular graphics: *PLATON* (Spek, 2009 ▶); software used to prepare material for publication: *SHELXTL* (Sheldrick, 2008 ▶), *PLATON* and *Mercury* (Macrae *et al.*, 2006 ▶).

## Supplementary Material

Crystal structure: contains datablocks global, I. DOI: 10.1107/S1600536810031703/kj2152sup1.cif

Structure factors: contains datablocks I. DOI: 10.1107/S1600536810031703/kj2152Isup2.hkl

Additional supplementary materials:  crystallographic information; 3D view; checkCIF report

## Figures and Tables

**Table 1:** Selected torsion angles (°)

C12—C13—C14—C31	93.88 (10)
C31—C14—C15—C16	−93.78 (10)

**Table 2:** Hydrogen-bond geometry (Å, °)

*D*—H⋯*A*	*D*—H	H⋯*A*	*D*⋯*A*	*D*—H⋯*A*
N11—H11⋯O24^i^	0.906 (17)	1.942 (17)	2.8444 (12)	173.6 (15)

Symmetry code: (i) 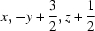.

**Fig. 1.**
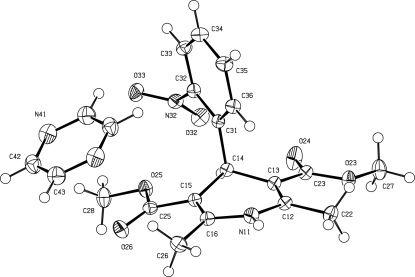
The asymmetric unit of the title compound, with the atom labeling scheme and 50% probability displacement ellipsoids.

**Fig. 2.**
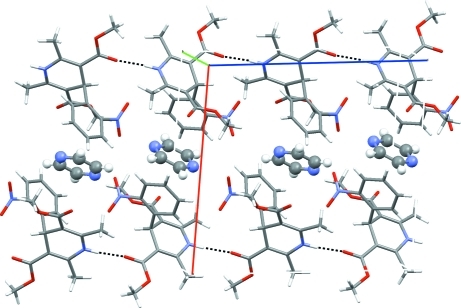
View down the b axis displaying the hydrogen bonding (black-dashed lines) between nifedipine molecules. The pyrazine molecules (ball-and-stick mode) are positioned between the one-dimensional nifedipine rows (right). The direction of the a axis is the red line, the b axis is green, and the c axis is blue.

## supplementary crystallographic information

### Comment

Designing, preparing, and characterizing cocrystalline materials is a rapidly growing area of research, especially in the area of pharmaceutics, due to their ability to alter the physicochemical properties of active pharmaceutical ingredients (APIs) (Schultheiss *et al.*, 2009) and provide drug repositioning or life-cycle management (Trask, 2007). Cocrystals are multi-component crystals where the individual, neutral molecules are typically held together through hydrogen-bonding. Nifedipine (1,4-dihydro-2,6-dimethyl-4-(2-nitrophenyl) -3,5-pyridine dicarboxylic acid dimethyl ester),a calcium-channel blocker, is known to exist in a variety of crystalline forms: polymorphs (Burger *et al.*, 1996), solvates/hydrates (Caira *et al.*, 2003), and a metal complex (Bontchev *et al.*, 2003), as well as a non-crystalline, amorphous phase (Miyazaki *et al.*, 2007). Suprisingly, examples of nifedipine cocrystals have yet to be published in the open literature, and thus we report here the 2:1 cocrystal of nifedipine and pyrazine.

A view of the asymmetric unit of the title compound and its numbering scheme are displayed in Fig. 1. The material crystallizes in a 2:1 (nifedipine:pyrazine) stoichiometric ratio, although the asymmetric unit contains the components in a 1:0.5 ratio, because the center of the pyrazine ring resides on an inversion center. It should also be noted that the nitro-substituted phenyl ring is relatively orthogonal ("axial") to the dihydropyridine ring (Table 1) which is displayed in Fig. 1. Nonetheless, the nifedipine molecules are linked into linear, one-dimensional chains with a graph set notation of C(6) through N—H···O hydrogen bonds from the N—H moiety to a carbonyl moiety, Table 2. The hydrogen bonds are running along the crystallographic *c* axis. Interestingly, the pyrazine molecules are not participating in hydrogen bonding with nifedipine, but are organized in between nifedipine rows through multiple van der Waals interactions (Fig. 2). Upon extending the structure into three-dimensions, the organization of the pyrazine molecules within the crystal structure are clearly shown. The pyrazine molecules are not only between one-dimensional rows of nifedipine, but also 'sandwiched' between methyl-ester groups from neighboring nifedipine molecules.

### Experimental

The title compound was prepared by adding solid nifedipine to a nearly saturated solution of pyrazine in methanol and allowed to stir for ~24 h at ambient temperature before filtering. Crystals of suitable size for single-crystal analysis were obtained directly from the experiment.

### Refinement

The amino H-atom was located in a difference Fourier map. All other H-atoms were positioned geometrically and allowed to ride on their parent atoms with U(H) set to 1.5U_eq_(C) for methyl and 1.2U_eq_(C) for all other carbon atoms.

### Crystal data

**Table:**

C_19_H_20_N_3_O_6_	*F*(000) = 812
*M**_r_* = 386.38	*D*_x_ = 1.429 Mg m^−^^3^
Monoclinic, *P*2_1_/*c*	Mo *K*α radiation, λ = 0.71073 Å
Hall symbol: -P 2ybc	Cell parameters from 9767 reflections
*a* = 13.6278 (14) Å	θ = 2.6–31.7°
*b* = 9.1594 (9) Å	µ = 0.11 mm^−^^1^
*c* = 14.4432 (14) Å	*T* = 120 K
β = 94.841 (4)°	Prism, colourless
*V* = 1796.4 (3) Å^3^	0.24 × 0.18 × 0.10 mm
*Z* = 4	

### Data collection

**Table:**

Bruker APEXII CCD diffractometer	4916 reflections with *I* > 2σ(*I*)
Radiation source: fine-focus sealed tube	*R*_int_ = 0.036
graphite	θ_max_ = 31.8°, θ_min_ = 2.6°
φ and ω scans	*h* = −20→19
27572 measured reflections	*k* = −13→13
6070 independent reflections	*l* = −17→21

### Refinement

**Table:**

Refinement on *F*^2^	Primary atom site location: structure-invariant direct methods
Least-squares matrix: full	Secondary atom site location: difference Fourier map
*R*[*F*^2^ > 2σ(*F*^2^)] = 0.043	Hydrogen site location: inferred from neighbouring sites
*wR*(*F*^2^) = 0.125	H atoms treated by a mixture of independent and constrained refinement
*S* = 1.07	*w* = 1/[σ^2^(*F*_o_^2^) + (0.070*P*)^2^ + 0.250*P*] where *P* = (*F*_o_^2^ + 2*F*_c_^2^)/3
6070 reflections	(Δ/σ)_max_ < 0.001
261 parameters	Δρ_max_ = 0.48 e Å^−^^3^
0 restraints	Δρ_min_ = −0.24 e Å^−^^3^

### Special details

**Table:**

Geometry. All e.s.d.'s (except the e.s.d. in the dihedral angle between two l.s. planes) are estimated using the full covariance matrix. The cell e.s.d.'s are taken into account individually in the estimation of e.s.d.'s in distances, angles and torsion angles; correlations between e.s.d.'s in cell parameters are only used when they are defined by crystal symmetry. An approximate (isotropic) treatment of cell e.s.d.'s is used for estimating e.s.d.'s involving l.s. planes.
Refinement. Refinement of *F*^2^ against ALL reflections. The weighted *R*-factor *wR* and goodness of fit *S* are based on *F*^2^, conventional *R*-factors *R* are based on *F*, with *F* set to zero for negative *F*^2^. The threshold expression of *F*^2^ > σ(*F*^2^) is used only for calculating *R*-factors(gt) *etc*. and is not relevant to the choice of reflections for refinement. *R*-factors based on *F*^2^ are statistically about twice as large as those based on *F*, and *R*- factors based on ALL data will be even larger.

### Fractional atomic coordinates and isotropic or equivalent isotropic displacement parameters (Å^2^)

**Table:**

	*x*	*y*	*z*	*U*_iso_*/*U*_eq_	
N11	0.91012 (7)	0.66064 (10)	0.58015 (6)	0.01904 (17)	
H11	0.9275 (12)	0.6672 (17)	0.6420 (12)	0.033 (4)*	
C12	0.96337 (7)	0.73800 (11)	0.52067 (6)	0.01680 (18)	
C13	0.92963 (7)	0.74418 (11)	0.42914 (6)	0.01588 (17)	
C14	0.82937 (7)	0.68380 (10)	0.39640 (6)	0.01536 (17)	
H14	0.8323	0.6431	0.3324	0.018*	
C15	0.80215 (7)	0.56174 (10)	0.46039 (6)	0.01635 (17)	
C16	0.83786 (7)	0.56204 (11)	0.55095 (7)	0.01793 (18)	
C22	1.05495 (8)	0.80615 (12)	0.56668 (7)	0.0214 (2)	
H22A	1.0559	0.9104	0.5515	0.032*	
H22B	1.1129	0.7586	0.5444	0.032*	
H22C	1.0558	0.7940	0.6342	0.032*	
C23	0.98313 (7)	0.80823 (11)	0.35653 (7)	0.01736 (18)	
O23	1.06719 (5)	0.87616 (9)	0.38444 (5)	0.02116 (16)	
O24	0.95391 (6)	0.80104 (11)	0.27468 (5)	0.0315 (2)	
C25	0.73593 (7)	0.44528 (11)	0.42414 (7)	0.01813 (18)	
O25	0.71443 (6)	0.45992 (8)	0.33133 (5)	0.02199 (16)	
O26	0.70447 (6)	0.34517 (9)	0.46697 (6)	0.02589 (17)	
C26	0.80877 (9)	0.46176 (12)	0.62596 (7)	0.0237 (2)	
H26A	0.7373	0.4660	0.6291	0.036*	
H26B	0.8418	0.4921	0.6858	0.036*	
H26C	0.8282	0.3616	0.6121	0.036*	
C27	1.11954 (9)	0.93700 (14)	0.31148 (8)	0.0264 (2)	
H27A	1.1818	0.9789	0.3380	0.040*	
H27B	1.0795	1.0136	0.2795	0.040*	
H27C	1.1330	0.8601	0.2671	0.040*	
C28	0.65577 (9)	0.34450 (13)	0.28780 (8)	0.0276 (2)	
H28A	0.6401	0.3676	0.2219	0.041*	
H28B	0.5946	0.3347	0.3184	0.041*	
H28C	0.6926	0.2526	0.2934	0.041*	
C31	0.75049 (7)	0.80317 (10)	0.39303 (6)	0.01571 (17)	
C32	0.67448 (7)	0.82130 (11)	0.32317 (7)	0.01755 (18)	
N32	0.66655 (7)	0.73025 (10)	0.23900 (6)	0.01976 (17)	
O32	0.73861 (6)	0.71650 (9)	0.19529 (5)	0.02539 (17)	
O33	0.58615 (6)	0.67540 (9)	0.21593 (6)	0.02713 (18)	
C33	0.60043 (8)	0.92431 (12)	0.32730 (7)	0.0219 (2)	
H33	0.5497	0.9320	0.2782	0.026*	
C34	0.60109 (8)	1.01542 (12)	0.40326 (8)	0.0239 (2)	
H34	0.5512	1.0872	0.4068	0.029*	
C35	0.67529 (8)	1.00121 (12)	0.47455 (7)	0.0227 (2)	
H35	0.6763	1.0634	0.5273	0.027*	
C36	0.74769 (8)	0.89694 (11)	0.46907 (7)	0.01926 (19)	
H36	0.7975	0.8886	0.5189	0.023*	
N41	0.45265 (8)	0.46068 (12)	0.41368 (7)	0.0302 (2)	
C42	0.45577 (9)	0.37242 (13)	0.48684 (9)	0.0289 (2)	
H42	0.4248	0.2796	0.4805	0.035*	
C43	0.50253 (9)	0.41122 (14)	0.57190 (8)	0.0297 (2)	
H43	0.5027	0.3439	0.6220	0.036*	

### Atomic displacement parameters (Å^2^)

**Table:**

	*U*^11^	*U*^22^	*U*^33^	*U*^12^	*U*^13^	*U*^23^
N11	0.0221 (4)	0.0250 (4)	0.0100 (3)	−0.0011 (3)	0.0008 (3)	0.0011 (3)
C12	0.0176 (4)	0.0209 (4)	0.0119 (4)	0.0010 (3)	0.0011 (3)	−0.0001 (3)
C13	0.0159 (4)	0.0201 (4)	0.0116 (4)	−0.0002 (3)	0.0012 (3)	0.0004 (3)
C14	0.0169 (4)	0.0183 (4)	0.0109 (4)	−0.0003 (3)	0.0015 (3)	−0.0002 (3)
C15	0.0170 (4)	0.0175 (4)	0.0147 (4)	0.0003 (3)	0.0025 (3)	0.0005 (3)
C16	0.0199 (4)	0.0197 (4)	0.0145 (4)	0.0015 (3)	0.0032 (3)	0.0013 (3)
C22	0.0200 (5)	0.0293 (5)	0.0143 (4)	−0.0021 (4)	−0.0014 (3)	−0.0011 (4)
C23	0.0174 (4)	0.0215 (4)	0.0132 (4)	0.0005 (3)	0.0014 (3)	−0.0002 (3)
O23	0.0211 (3)	0.0285 (4)	0.0141 (3)	−0.0065 (3)	0.0031 (3)	−0.0004 (3)
O24	0.0279 (4)	0.0550 (6)	0.0111 (3)	−0.0138 (4)	−0.0004 (3)	0.0043 (3)
C25	0.0182 (4)	0.0187 (4)	0.0178 (4)	0.0024 (3)	0.0029 (3)	−0.0004 (3)
O25	0.0246 (4)	0.0240 (4)	0.0170 (3)	−0.0059 (3)	−0.0004 (3)	−0.0019 (3)
O26	0.0308 (4)	0.0224 (4)	0.0247 (4)	−0.0055 (3)	0.0037 (3)	0.0025 (3)
C26	0.0300 (5)	0.0242 (5)	0.0173 (4)	−0.0007 (4)	0.0043 (4)	0.0057 (4)
C27	0.0270 (5)	0.0335 (6)	0.0196 (5)	−0.0097 (4)	0.0066 (4)	0.0014 (4)
C28	0.0273 (5)	0.0295 (5)	0.0256 (5)	−0.0089 (4)	0.0003 (4)	−0.0067 (4)
C31	0.0166 (4)	0.0175 (4)	0.0131 (4)	−0.0010 (3)	0.0018 (3)	0.0015 (3)
C32	0.0188 (4)	0.0202 (4)	0.0135 (4)	−0.0018 (3)	0.0001 (3)	0.0002 (3)
N32	0.0221 (4)	0.0224 (4)	0.0142 (4)	0.0003 (3)	−0.0022 (3)	0.0010 (3)
O32	0.0270 (4)	0.0339 (4)	0.0155 (3)	0.0015 (3)	0.0033 (3)	−0.0017 (3)
O33	0.0249 (4)	0.0304 (4)	0.0245 (4)	−0.0054 (3)	−0.0067 (3)	−0.0025 (3)
C33	0.0204 (5)	0.0244 (5)	0.0204 (5)	0.0022 (4)	−0.0012 (3)	0.0022 (4)
C34	0.0252 (5)	0.0227 (5)	0.0237 (5)	0.0054 (4)	0.0014 (4)	0.0010 (4)
C35	0.0276 (5)	0.0208 (4)	0.0197 (5)	0.0031 (4)	0.0017 (4)	−0.0029 (4)
C36	0.0223 (5)	0.0204 (4)	0.0149 (4)	0.0009 (3)	0.0002 (3)	−0.0008 (3)
N41	0.0291 (5)	0.0373 (5)	0.0247 (5)	−0.0013 (4)	0.0058 (4)	−0.0037 (4)
C42	0.0283 (6)	0.0271 (5)	0.0327 (6)	−0.0038 (4)	0.0101 (4)	−0.0035 (5)
C43	0.0313 (6)	0.0325 (6)	0.0264 (5)	0.0000 (5)	0.0093 (4)	0.0046 (5)

### Geometric parameters (Å, °)

**Table:**

N11—C12	1.3682 (13)	C27—H27A	0.9800
N11—C16	1.3759 (13)	C27—H27B	0.9800
N11—H11	0.906 (17)	C27—H27C	0.9800
C12—C13	1.3636 (13)	C28—H28A	0.9800
C12—C22	1.4996 (14)	C28—H28B	0.9800
C13—C23	1.4507 (13)	C28—H28C	0.9800
C13—C14	1.5125 (13)	C31—C32	1.3937 (13)
C14—C15	1.5165 (13)	C31—C36	1.3973 (13)
C14—C31	1.5311 (13)	C32—C33	1.3865 (14)
C14—H14	1.0000	C32—N32	1.4706 (13)
C15—C16	1.3566 (13)	N32—O32	1.2180 (12)
C15—C25	1.4651 (14)	N32—O33	1.2257 (12)
C16—C26	1.4989 (14)	C33—C34	1.3778 (15)
C22—H22A	0.9800	C33—H33	0.9500
C22—H22B	0.9800	C34—C35	1.3871 (15)
C22—H22C	0.9800	C34—H34	0.9500
C23—O24	1.2170 (12)	C35—C36	1.3802 (14)
C23—O23	1.3357 (12)	C35—H35	0.9500
O23—C27	1.4342 (12)	C36—H36	0.9500
C25—O26	1.2050 (12)	N41—C42	1.3282 (17)
C25—O25	1.3544 (12)	N41—C43^i^	1.3311 (17)
O25—C28	1.4377 (13)	C42—C43	1.3819 (18)
C26—H26A	0.9800	C42—H42	0.9500
C26—H26B	0.9800	C43—N41^i^	1.3311 (17)
C26—H26C	0.9800	C43—H43	0.9500
			
C12—N11—C16	123.46 (8)	O23—C27—H27B	109.5
C12—N11—H11	118.4 (10)	H27A—C27—H27B	109.5
C16—N11—H11	117.8 (10)	O23—C27—H27C	109.5
C13—C12—N11	118.48 (9)	H27A—C27—H27C	109.5
C13—C12—C22	127.75 (9)	H27B—C27—H27C	109.5
N11—C12—C22	113.77 (8)	O25—C28—H28A	109.5
C12—C13—C23	124.66 (9)	O25—C28—H28B	109.5
C12—C13—C14	120.65 (8)	H28A—C28—H28B	109.5
C23—C13—C14	114.68 (8)	O25—C28—H28C	109.5
C13—C14—C15	109.88 (8)	H28A—C28—H28C	109.5
C13—C14—C31	111.23 (8)	H28B—C28—H28C	109.5
C15—C14—C31	109.79 (8)	C32—C31—C36	115.33 (9)
C13—C14—H14	108.6	C32—C31—C14	125.80 (8)
C15—C14—H14	108.6	C36—C31—C14	118.66 (8)
C31—C14—H14	108.6	C33—C32—C31	123.31 (9)
C16—C15—C25	120.41 (9)	C33—C32—N32	114.74 (9)
C16—C15—C14	119.99 (9)	C31—C32—N32	121.95 (9)
C25—C15—C14	119.60 (8)	O32—N32—O33	123.91 (9)
C15—C16—N11	119.08 (9)	O32—N32—C32	118.74 (9)
C15—C16—C26	126.89 (9)	O33—N32—C32	117.32 (9)
N11—C16—C26	114.01 (9)	C34—C33—C32	119.38 (10)
C12—C22—H22A	109.5	C34—C33—H33	120.3
C12—C22—H22B	109.5	C32—C33—H33	120.3
H22A—C22—H22B	109.5	C33—C34—C35	119.32 (10)
C12—C22—H22C	109.5	C33—C34—H34	120.3
H22A—C22—H22C	109.5	C35—C34—H34	120.3
H22B—C22—H22C	109.5	C36—C35—C34	120.14 (10)
O24—C23—O23	121.36 (9)	C36—C35—H35	119.9
O24—C23—C13	122.49 (9)	C34—C35—H35	119.9
O23—C23—C13	116.15 (8)	C35—C36—C31	122.51 (9)
C23—O23—C27	115.24 (8)	C35—C36—H36	118.7
O26—C25—O25	121.79 (9)	C31—C36—H36	118.7
O26—C25—C15	127.27 (9)	C42—N41—C43^i^	115.38 (11)
O25—C25—C15	110.91 (8)	C42—N41—C42^ii^	106.16 (7)
C25—O25—C28	115.21 (8)	C43^i^—N41—C42^ii^	137.11 (8)
C16—C26—H26A	109.5	N41—C42—C43	122.15 (11)
C16—C26—H26B	109.5	N41—C42—H42	118.9
H26A—C26—H26B	109.5	C43—C42—H42	118.9
C16—C26—H26C	109.5	N41^i^—C43—C42	122.46 (11)
H26A—C26—H26C	109.5	N41^i^—C43—H43	118.8
H26B—C26—H26C	109.5	C42—C43—H43	118.8
O23—C27—H27A	109.5		
			
C16—N11—C12—C13	15.45 (15)	C14—C15—C25—O26	−176.60 (10)
C16—N11—C12—C22	−164.07 (9)	C16—C15—C25—O25	−175.78 (9)
N11—C12—C13—C23	−173.05 (9)	C14—C15—C25—O25	5.23 (12)
C22—C12—C13—C23	6.40 (17)	O26—C25—O25—C28	−2.79 (14)
N11—C12—C13—C14	7.81 (14)	C15—C25—O25—C28	175.50 (8)
C22—C12—C13—C14	−172.75 (9)	C13—C14—C31—C32	138.67 (9)
C12—C13—C14—C15	−27.91 (12)	C15—C14—C31—C32	−99.50 (11)
C23—C13—C14—C15	152.87 (8)	C13—C14—C31—C36	−46.76 (11)
C12—C13—C14—C31	93.88 (10)	C15—C14—C31—C36	75.07 (11)
C23—C13—C14—C31	−85.35 (10)	C36—C31—C32—C33	−0.04 (14)
C13—C14—C15—C16	28.86 (12)	C14—C31—C32—C33	174.69 (9)
C31—C14—C15—C16	−93.78 (10)	C36—C31—C32—N32	−179.46 (9)
C13—C14—C15—C25	−152.15 (8)	C14—C31—C32—N32	−4.73 (15)
C31—C14—C15—C25	85.21 (10)	C33—C32—N32—O32	130.11 (10)
C25—C15—C16—N11	171.13 (9)	C31—C32—N32—O32	−50.42 (13)
C14—C15—C16—N11	−9.89 (14)	C33—C32—N32—O33	−48.13 (12)
C25—C15—C16—C26	−7.30 (15)	C31—C32—N32—O33	131.33 (10)
C14—C15—C16—C26	171.68 (9)	C31—C32—C33—C34	0.63 (16)
C12—N11—C16—C15	−14.39 (15)	N32—C32—C33—C34	−179.91 (9)
C12—N11—C16—C26	164.24 (9)	C32—C33—C34—C35	−0.67 (16)
C12—C13—C23—O24	173.91 (11)	C33—C34—C35—C36	0.14 (17)
C14—C13—C23—O24	−6.90 (14)	C34—C35—C36—C31	0.48 (16)
C12—C13—C23—O23	−6.01 (15)	C32—C31—C36—C35	−0.51 (14)
C14—C13—C23—O23	173.19 (8)	C14—C31—C36—C35	−175.64 (9)
O24—C23—O23—C27	−0.71 (15)	C43^i^—N41—C42—C43	−0.10 (19)
C13—C23—O23—C27	179.21 (9)	C42^ii^—N41—C42—C43	−169.14 (10)
C16—C15—C25—O26	2.38 (16)	N41—C42—C43—N41^i^	0.1 (2)

Symmetry codes: (i) −*x*+1, −*y*+1, −*z*+1; (ii) −*x*, −*y*, −*z*.

### Hydrogen-bond geometry (Å, °)

**Table:**

*D*—H···*A*	*D*—H	H···*A*	*D*···*A*	*D*—H···*A*
N11—H11···O24^iii^	0.906 (17)	1.942 (17)	2.8444 (12)	173.6 (15)

Symmetry codes: (iii) *x*, −*y*+3/2, *z*+1/2.
